# Trauma with Injury Severity Score of 75: Are These Unsurvivable Injuries?

**DOI:** 10.1371/journal.pone.0134821

**Published:** 2015-07-31

**Authors:** Jin Peng, Krista Wheeler, Junxin Shi, Jonathan Ira Groner, Kathryn Jo Haley, Huiyun Xiang

**Affiliations:** 1 Center for Pediatric Trauma Research, The Research Institute at Nationwide Children’s Hospital, Columbus, Ohio, United States of America; 2 The Ohio State University, College of Public Health, Columbus, Ohio, United States of America; 3 The Ohio State University, College of Medicine, Columbus, Ohio, United States of America; 4 Trauma Program, Department of Pediatric Surgery, Nationwide Children’s Hospital, Columbus, Ohio, United States of America; Azienda Ospedaliero-Universitaria Careggi, ITALY

## Abstract

Trauma patients with an ISS=75 have been deliberately excluded from some trauma studies because they were assumed to have "unsurvivable injuries." This study aimed to assess the true mortality among patients with an ISS=75, and to examine the characteristics and primary diagnoses of these patients. Retrospective review of the 2006-2010 U.S. Nationwide Emergency Department Sample (NEDS) generated 2,815 patients with an ISS=75 for analysis, representing an estimated 13,569 patients in the country. Dispositions from the emergency department and hospital for these patients were tabulated by trauma center level. Survivors and non-survivors were compared using Pearson's chi-square test. Primary diagnosis codes of these patients were tabulated by mortality status. Overall, about 48.6% of patients with an ISS=75 were discharged alive, 25.8% died and 25.6% had unknown mortality status. The mortality risks of these patients did not vary significantly across different levels of trauma centers (15.6% vs. 13.0%, P = 0.16). Non-survivors were more likely than survivors to: be male (81.2% vs. 74.4%, P < 0.0001), be over 65 years (20.3% vs. 10.2%, P < 0.0001), be uninsured (33.8% vs. 19.1%), have at least one chronic condition (58.0% vs. 43.7%, P <0.0001), sustain life-threatening injuries (79.2% vs. 49.4%, P<0.0001), sustain penetrating injuries (42.0% vs. 25.9%, P<0.0001), and have injuries caused by motor vehicle crashes (32.9% vs. 21.1%, P<0.0001) or firearms (21.9% vs. 4.4%, P<0.0001). The most frequent diagnosis code was 862.8 (injury to multiple and unspecified intrathoracic organs, without mention of open wound into cavity). Our results revealed that at least half of patients with an ISS=75 survived, demonstrating that the rationale for excluding patients with an ISS=75 from analysis is not always justified. To avoid bias and inaccurate results, trauma researchers should examine the mortality status of patients with an ISS=75 before exclusion, and explicitly describe their method of generating ISS scores.

## Introduction

Classification of injury by its severity is fundamental to injury research. A number of scales measuring injury severity have been developed since the late 1960s [1]. The Injury Severity Score (ISS), an anatomically based scale for rating the overall severity of multiple injuries, is one of the most commonly used scales in recent decades [2]. To calculate the ISS, the body is divided into six ISS body regions (Head, Face, Chest, Abdomen, Extremities, External). Each injured body region is assigned an Abbreviated Injury Scale (AIS) score ranging from 1 (minor injury) to 6 (maximum injury) [3]. The ISS score is then calculated as the sum of squares of each AIS score for the three most severely injured body regions. If any of the three AIS scores is a 6, the ISS score is automatically set as 75 [1]. AIS scores are assigned by trained coders who consult the medical record. Additionally, because of the widespread use of ISS scores, software programs (e.g. ICDMAP, ICDPIC) have been developed to translate the International Classification of Diseases, Ninth Revision, Clinical Modification (ICD-9-CM) diagnosis codes into injury severity scores.

Trauma patients with an ISS = 75 are often considered as sustaining the most severe injuries with the lowest possible survival rate among trauma researchers. However, our prior study on undertriage of major trauma patients indicated that a significant proportion of trauma patients with an ISS = 75 survived [4]. We found that a number of trauma studies excluded patients with an ISS = 75 from analysis without explicitly stating whether or not they examined the mortality status of these patients [5–9]. The exclusion of patients with an ISS = 75 can result in biases if most of these patients survived. Studies that evaluated the effects of interventions on improving trauma outcomes were at the highest risk of yielding biased results. Since patients with an ISS = 75 sustained the most severe injuries, interventions that performed well among patients with an ISS <75 might not be effective among patients with an ISS = 75. Therefore, by excluding patients with an ISS = 75, the effectiveness of those interventions may have been overestimated.

In this study, we aimed to assess the extent to which patients with an ISS = 75 seen at U.S. hospital-based EDs survived and to examine the characteristics and primary diagnoses of patients with an ISS = 75. We evaluated ISS = 75 when assigned by the International Classification of Diseases Program for Injury Categorization (ICDPIC), a statistical program that has been proven to be efficient in extracting injury severity scores from ICD-9-CM codes for large trauma datasets [10]. Our findings have important implications for trauma research quality improvement, especially as decisions are made about excluding patients with an ISS = 75.

## Methods

### Data Source and Study Population

The Nationwide Emergency Department Sample (NEDS) from 2006 to 2010 was used for analysis. Sampled from the State Emergency Department Databases (SEDD) and State Inpatient Databases (SID), the NEDS is the largest all-payer emergency department (ED) database in the U.S. [11] It contains information on ED visits that both result in an admission, and do not result in an admission (e.g., treat-and-release visits and transfers to another hospital). The NEDS contains data from 30 million discharges (representing 20% of all ED visits in the U.S.) each year, which can be weighted to produce national estimates of hospital-based ED visits. In this study, we used NEDS core files for demographic characteristics, ED disposition and ICD-9-CM diagnosis codes; the hospital files for hospital characteristics; and the inpatient files for inpatient disposition.

The study population was trauma patients with an ISS = 75 who had visited emergency departments (EDs) in the United States from January 1, 2006 to December 31, 2010. We searched all 15 possible diagnoses for each patient and identified trauma patients using ICD-9-CM diagnosis codes (800 to 959). To be consistent with the definition of a “trauma patient” used widely by trauma centers in the U.S., we excluded trauma patients who only had injuries from late effects (905 to 909), sprains and strains (840 to 848), superficial injuries (910–924) and injuries due to foreign bodies (930 to 939).

### Statistical Analysis

Data analysis was performed using SAS 9.3 software (SAS Institute, Cary, NC). The Injury Severity Score (ISS) and other injury-related variables (e.g. injury mechanism) were generated using STATA 13 (StataCorp, College Station, TX). The NEDS started using the ICD Programs for Injury Categorization (ICDPIC) to generate ISS values for each patient in 2009. The ICDPIC is a publicly available STATA program that translates ICD-9-CM diagnosis codes into AIS 90 and other injury scores [12], and we used this to generate the ISS and other injury-related variables for 2006–2008 NEDS. We then extracted all trauma patients with an ISS = 75 for the years 2006–2010. We also created a new variable that indicated life-threatening injuries using criteria from the American College of Surgeon Committee on Trauma (ACS-COT) (see S1 Appendix) [13]. Using the weighting variables provided in the NEDS, we produced national estimates of ED visits for trauma patients with an ISS = 75. We tabulated the patient and hospital characteristics of trauma patients with an ISS = 75. We also tabulated ED and hospital disposition by trauma center level. We expected that rates of transfer to short-term hospitals from non-trauma centers would be much higher than from trauma centers. The distributions of ED and hospital disposition across different levels of trauma centers were compared using Pearson’s chi-square test. We then divided patients with an ISS = 75 into three groups according to their mortality status. The first group included patients with an ISS = 75 who died either in the ED or in an inpatient unit. The second group consisted of patients with an ISS = 75 who were discharged alive, including patients who were treated and released from the ED or from an inpatient unit, discharged without treatment, or sent to home health care. The third group consisted of patients with an ISS = 75 with unknown mortality status, including patients who were transferred to a short-term hospital or elsewhere (e.g. skilled nursing facility), those who were discharged against medical advice, and those who were not admitted to the hospital but had an unknown destination. We then examined the differences between patients who died and those who were discharged alive by patient and hospital characteristics, using Pearson’s chi-square test. We tabulated the frequency of major ICD-9-CM diagnosis codes by mortality status. The major ICD-9-CM code was identified as the first diagnosis code (out of 15 diagnosis codes) that was assigned by ICDPIC with an AIS = 6. Six patients were excluded from this analysis because they did not have an injury of AIS = 6 (an ISS of 75 is not only given to patients who have a single injury of AIS = 6 but also to those who have three injuries of AIS = 5).

This study was reviewed and considered exempt by the Institutional Review Board of Nationwide Children’s Hospital, because we analyzed publicly available data with all personal identifiers removed.

## Results

### Patient characteristics

A nationally representative sample of 2,815 patients with an ISS = 75 was identified in our study. It represented approximately 20% of all patients with an ISS = 75 (13,569) seen at U.S. hospital-based EDs between 2006 and 2010. The average patient age was 40.5 years, with the majority between18 to54 years old (63.7%). Pediatric patients and older adults were 11.6% and 24.7% of the sample, respectively. Most patients were males (76.3%) and most patients lived in metropolitan areas (78.2%). Over half had chronic conditions (53.7%). The majority sustained ACS-COT defined life-threatening injuries (see S1 Appendix) (62.7%), with blunt injuries (72.1%) being more common than penetrating injuries. Private insurance was the most common type of primary payer (35.7%). Motor vehicle traffic was the most common type of injury mechanism (28.3%) (Table 1). Most patients were treated at metropolitan teaching hospitals (55.8%), and about 41.4% were treated at non-trauma hospitals or level III trauma centers (Table 1).

**Table 1:** Patient and hospital characteristics of trauma patients with ISS 75, NEDS 2006–2010.

Variable	Sample (n = 2815)	National estimates (n = 13569)	Col %
Patient-Level Characteristics			
Gender^d^			
Male	2133	10344	76.3%
Female	678	3209	23.7%
Age group^d^			
<18 years old	334	1573	11.6%
18–34 years old	917	4477	33.0%
35–54 years old	866	4165	30.7%
55–64 years old	269	1324	9.8%
> = 65 years old	426	2015	14.9%
Patient's residence location^d^			
Large central metropolitan	817	3894	29.3%
Large fringe metropolitan	580	2723	20.5%
Medium metropolitan	515	2369	17.8%
Small metropolitan	264	1411	10.6%
Micropolitan	318	1589	12.0%
Not metropolitan or micropolitan	268	1307	9.8%
Median household income quartiles^a^ ^,^ ^d^			
1st quartile (Lowest)	888	4245	32.7%
2nd quartile	737	3617	27.9%
3rd quartile	612	2912	22.4%
4th quartile (Highest)	463	2202	17.0%
Primary expected payer^d^			
Medicare	390	1825	13.6%
Medicaid	444	2138	15.9%
Private including HMO	980	4792	35.7%
Self-pay	588	2811	20.9%
Other	382	1872	13.9%
Chronic conditions			
No chronic conditions	1306	6279	46.3%
At least one chronic condition	1509	7291	53.7%
Life-threatening injury^b^			
Not life-threatening	1077	5066	37.3%
Life-threatening	1738	8503	62.7%
Blunt/Penetrating injury^d^			
Blunt injury	1549	7456	72.1%
Penetrating injury	602	2884	27.9%
Injury mechanism^d^			
Motor vehicle traffic	703	3418	28.3%
Firearm	236	1155	9.5%
Cut/pierce	366	1729	14.3%
Fall	326	1577	13.0%
Struck by, against	263	1229	10.2%
Other categories	612	2988	24.7%
Hospital-Level Characteristics			
Hospital type			
Metropolitan non-teaching	860	3948	29.1%
Metropolitan teaching	1570	7566	55.8%
Non-metropolitan	385	2055	15.1%
Hospital region			
Northeast	504	2313	17.0%
Midwest	695	3482	25.7%
South	951	4433	32.7%
West	665	3341	24.6%
Trauma center designation			
Non-trauma or level III trauma centers	1198	5617	41.4%
Level I or level II trauma centers	1052	5026	37.0%
Other collapsed categories^c^	565	2926	21.6%

Abbreviations: NEDS, Nationwide Emergency Department Sample.

^a.^ National quartiles for median household income of patient's home ZIP code.

^b.^ Life-threatening injuries were defined by American College of Surgeons Committee on Trauma (ACS-COT).

^c.^ Other collapsed categories included trauma level I or II collapsed category and trauma level I, II, or III collapsed category.

^d.^ Variables had missing observations.

### Emergency department and hospital disposition by hospital trauma level

The emergency department (ED) and hospital dispositions among patients with an ISS = 75 differed significantly between lower and higher level of trauma centers (Table 2). Specifically, patients seen at the ED of non-trauma hospitals or level III trauma centers were more likely than those seen at level I or II trauma centers to be routinely discharged (46.1% vs. 14.5%, P < 0.0001), or to be transferred to another hospital (13.9% vs. 0.9%, P < 0.0001). Although patients with an ISS = 75 seen at non-trauma hospitals or level III trauma centers appeared to be at higher risk of mortality, the proportions of patients that died in the ED did not vary significantly between lower level trauma centers and higher level trauma centers (15.6% vs. 13.0%, P = 0.16). Patients with an ISS = 75 admitted as an inpatient at non-trauma hospitals or level III trauma centers were more likely than those admitted to level I or II trauma centers to then be routinely discharged from the hospital (46.6% vs. 35.7%, P = 0.02) (Table 2).

**Table 2:** Disposition distribution among patients with ISS of 75, NEDS 2006–2010^a^.

Disposition	Non-trauma or level III trauma centers			Level I or level II trauma centers			
	Sample	National estimates	Col %	Sample	National estimates	Col %	P-value
ED disposition							
Routine^b^	576	2586	46.1%	158	728	14.5%	<.0001
Admitted as inpatient to the same hospital	245	1169	20.8%	712	3443	68.5%	<.0001
Died in the ED	174	878	15.6%	141	653	13.0%	0.16
Transfer to short-term hospital	158	780	13.9%	<10	45	0.9%	<.0001
Other transfers^c^	22	94	1.7%	<10	<10	0.2%	0.01
Not admitted, destination unknown	17	73	1.3%	23	116	2.3%	0.52
Against medical advice	<10	17	0.3%	<10	18	0.3%	0.86
Home health care	<10	14	0.2%	<10	14	0.3%	0.93
Discharged alive, destination unknown	<10	<10	0.1%	N/A	N/A	N/A	N/A
Total	1198	5617	100.0%	1052	5026	100.0%	N/A
Inpatient disposition							
Routine^b^	116	545	46.6%	256	1230	35.7%	0.02
Other transfers^c^	58	289	24.7%	219	1075	31.2%	0.11
Died in the hospital	45	200	17.1%	164	801	23.3%	0.05
Home health care	12	60	5.2%	28	126	3.7%	0.33
Transfer to short-term hospital	10	48	4.1%	43	203	5.9%	0.33
Against medical advice	<10	15	1.3%	<10	<10	0.1%	<.0001
Discharged alive, destination unknown	<10	12	1.0%	<10	<10	0.1%	<.0001
Total	245	1169	100.0%	712	3443	100.0%	N/A

Abbreviations: NEDS, Nationwide Emergency Department Sample; ED, Emergency Department.

^a.^ Data from other collapsed trauma center categories were not shown in the table.

^b.^ Routine indicated that patients were treated and released from the hospital.

^c.^ Included skilled nursing facility, intermediate care and another type of facility.

### Comparison of survivors and non-survivors

Among patients with an ISS = 75, 48.6% were discharged alive (survivors), 25.8% died (non-survivors) and 25.6% had unknown mortality status. Non-survivors were more likely than survivors to be male (81.2% vs. 74.4%, P < 0.0001), be above 65 years (20.3% vs. 10.2%, P < 0.0001), be uninsured (33.8% vs. 19.1%), have at least one chronic condition (58.0% vs. 43.7%, P <0.0001), sustain life-threatening injuries (79.2% vs. 49.4%, P<0.0001), sustain penetrating injuries (42.0% vs. 25.9%, P<0.0001), and have injuries caused by motor vehicle traffic (32.9% vs. 21.1%, P<0.0001) or firearm (21.9% vs. 4.4%, P<0.0001). Non-survivors were also more likely to be seen at metropolitan teaching hospitals (60.7% vs. 52.5%, P = 0.01), and be seen at level I or II trauma centers (41.6% vs. 31.8%, P < 0.0001) (Table 3). Non-survivors and survivors did not differ significantly in patients’ residence location, median household income, and hospital region (Table 3).

**Table 3:** Patient and hospital characteristics of patients with ISS 75 by mortality status, NEDS 2006–2010^a^ ^.^.

Variable	Died^b^			Discharged alive^c^			P-value
	Sample (n = 711)	National estimates (n = 3496)	Col %	Sample (n = 1404)	National estimates (n = 6602)	Col %	
Patient-Level Characteristics							
Gender							
Male	572	2835	81.2%	1037	4905	74.4%	
Female	138	657	18.8%	365	1688	25.6%	<.0001
Age group							
<18 years old	61	286	8.2%	207	967	14.6%	<.0001
18–34 years old	223	1135	32.5%	461	2200	33.3%	0.72
35–54 years old	193	937	26.8%	464	2177	33.0%	0.01
55–64 years old	86	427	12.2%	123	586	8.9%	0.03
> = 65 years old	148	711	20.3%	149	672	10.2%	<.0001
Patient's residence location							
Large central metropolitan	237	1116	33.3%	391	1865	28.6%	0.07
Large fringe metropolitan	120	592	17.6%	300	1350	20.7%	0.24
Medium metropolitan	141	665	19.8%	257	1143	17.5%	0.29
Small metropolitan	58	321	9.6%	134	697	10.7%	0.50
Micropolitan	75	386	11.5%	161	780	12.0%	0.81
Not metropolitan or micropolitan	56	275	8.2%	144	687	10.5%	0.16
Median household income quartiles^d^							
1st quartile (lowest)	244	1194	36.2%	450	2097	32.8%	0.19
2nd quartile	192	982	29.8%	361	1703	26.6%	0.13
3rd quartile	135	635	19.3%	317	1486	23.2%	0.05
4th quartile (highest)	103	485	14.7%	231	1108	17.3%	0.20
Primary expected payer							
Medicare	114	542	15.6%	160	719	11.0%	0.01
Medicaid	83	406	11.6%	221	1042	16.0%	0.01
Private including HMO	218	1059	30.4%	485	2322	35.6%	0.02
Self-pay	232	1178	33.8%	273	1247	19.1%	<.0001
Other	61	298	8.5%	245	1189	18.2%	<.0001
Chronic conditions							
No chronic conditions	290	1469	42.0%	792	3717	56.3%	
At least one chronic condition	421	2027	58.0%	612	2885	43.7%	<.0001
Life-threatening injuries^e^							
Not life-threatening	147	726	20.8%	722	3342	50.6%	
Life-threatening	564	2769	79.2%	682	3260	49.4%	<.0001
Blunt/Penetrating injuries							
Blunt injuries	358	1793	58.0%	726	3373	74.1%	
Penetrating injuries	269	1299	42.0%	247	1179	25.9%	<.0001
Injury Mechanism							
Motor vehicle traffic	217	1088	32.9%	262	1214	21.1%	<.0001
Firearm	148	723	21.9%	50	250	4.4%	<.0001
Cut/pierce	121	576	17.4%	197	928	16.2%	0.52
Fall	69	356	10.8%	129	586	10.2%	0.80
Struck by, against	32	153	4.6%	182	851	14.8%	<.0001
Other categories	84	411	12.4%	400	1914	33.3%	<.0001
Hospital-Level Characteristics							
Hospital type							
Metropolitan non-teaching	190	878	25.1%	471	2103	31.9%	0.01
Metropolitan teaching	436	2121	60.7%	726	3464	52.5%	0.01
Non-metropolitan	85	497	14.2%	207	1035	15.7%	0.52
Hospital region							
Northeast	128	592	16.9%	243	1108	16.8%	0.96
Midwest	145	728	20.8%	329	1620	24.5%	0.12
South	248	1213	34.7%	494	2204	33.4%	0.63
West	190	964	27.6%	338	1670	25.3%	0.39
Trauma Center Designation							
Non-trauma or level III trauma centers	219	1078	30.8%	709	3223	48.8%	<.0001
Level I or level II trauma centers	305	1455	41.6%	446	2102	31.8%	<.0001
Other categories	187	963	27.6%	249	1277	19.3%	<.0001

Abbreviations: NEDS, Nationwide Emergency Department Sample.

^a.^ Data on patients with unknown mortality status were not shown in the table.

^b.^ Includes patients who died in the ED and those who died in the hospital.

^c.^ Includes patients who were treated and released, discharged alive or sent to home health care from the ED, and patients who were treated and released, discharged alive or sent to home health care from the hospital.

^d.^ National quartiles for median household income of patient's home ZIP code.

^e.^ Life-threatening injuries were defined by American College of Surgeons Committee on Trauma (ACS-COT).

### Frequently occurring primary diagnosis codes

There were six frequently occurring primary diagnosis codes: 862.8 (Injury to multiple and unspecified intrathoracic organs, without mention of open wound into cavity), 861.13 (Laceration of heart with penetration of heart chambers with open wound into thorax), 806.01 (Closed fracture of C1–C4 level with complete lesion of cord), 929.9 (Crushing injury of unspecified site), 926.8 (Crushing injury of multiple sites of trunk) and 952.01(C1–C4 level with complete lesion of spinal cord) (Table 4). A significant proportion of patients with certain diagnosis codes survived. Specifically, 86.8% of patients with diagnosis code 929.9 survived, 68.6% of patients with diagnosis code 926.8 survived, and 55.1% of patients with diagnosis code 862.8 survived (Table 4).

**Table 4:** ISS 75 patients’ primary diagnosis codes by mortality status, NEDS 2006–2010 ^a^.

ICD-9-CM Code	Died^b^			Discharged alive^c^			Unknown mortality status^d^			Total		
	Sample	National estimates	Row %	Sample	National estimates	Row %	Sample	National estimates	Row %	Sample	National estimates	Row %
862.8^e^	243	1237	22.2%	667	3069	55.1%	259	1261	22.7%	1169	5567	100.0%
861.13^e^	272	1311	44.2%	274	1326	44.7%	69	327	11.0%	615	2964	100.0%
806.01^e^	121	597	32.3%	27	123	6.7%	224	1126	61.0%	372	1846	100.0%
929.9^e^	12	55	3.7%	274	1304	86.8%	29	144	9.6%	315	1503	100.0%
926.8^e^	19	91	9.0%	143	692	68.6%	41	226	22.4%	203	1009	100.0%
952.01^e^	30	143	31.4%	<10	34	7.5%	56	279	61.2%	94	456	100.0%
Others	12	51	26.4%	11	54	28.0%	18	88	45.6%	41	193	100.0%
Total	709	3485	25.7%	1404	6602	48.8%	696	3452	25.5%	2809	13539	100.0%

Abbreviations: NEDS, Nationwide Emergency Department Sample; ICD-9-CM, International Classification of Diseases, Ninth Revision, Clinical Modification.

^a.^ Patients who had three AIS = 5 were excluded from the analysis.

^b.^ Includes patients who died in the ED and those who died in the hospital.

^c.^ Includes patients who were treated and released, discharged alive or sent to home health care from the ED, and patients who were treated and released, discharged alive or sent to home health care from the hospital.

^d.^ Includes patients who were transferred to short-term hospital or other transfers (e.g. skilled nursing facility) from the ED, those who were against medical advice in the ED, those who were not admitted to the hospital but with unknown destination, those who were transferred to short-term hospital or other transfers (e.g. skilled nursing facility) from the hospital and those who were against medical advice in the hospital.

^e.^ 862.8—Injury to multiple and unspecified intrathoracic organs, without mention of open wound into cavity.

861.13—Laceration of heart with penetration of heart chambers with open wound into thorax.

806.01—Closed fracture of C1–C4 level with complete lesion of cord.

929.9—Crushing injury of unspecified site.

926.8—Crushing injury of multiple sites of trunk.

952.01—C1–C4 level with complete lesion of spinal cord.

## Discussion

Ours is the first study to focus on the mortality status of patients with an ISS = 75 who visited U.S. hospital-based emergency departments (EDs). Results of our study indicate that almost half of patients with an ISS = 75 survived their injuries. Non-survivors were more likely than survivors to be male, be above 65 years, be uninsured, have at least one chronic condition, sustain life-threatening injuries and penetrating injuries, and have injuries caused by motor vehicle traffic or firearm. The most frequently occurring ICD-9 diagnosis code was 862.8 (Injury to multiple and unspecified intrathoracic organs, without mention of open wound into cavity).

According to the Association for the Advancement of Automotive Medicine (the parent body of the AIS), an AIS 6 (ISS = 75) is defined as the maximal injury, but this is not equivalent to death [14]. Although an ISS = 75 does not equate with death, it is expected that those patients with an ISS = 75 sustain the most severe injuries with the lowest possible survival rate. The relatively high survival rate among patients with an ISS = 75 could be attributed to the limitations of using computer-based ISS scoring. Some of those with an AIS = 6 in the NEDS survey probably did not deserve an AIS of 6. The STATA-ICDPIC program, which we used to generate AIS/ISS scores, may have overestimated the injury severities of patients with certain ICD-9 diagnosis codes (e.g. 929.9, 926.8, 862.8). Specifically, 46.5% of patients who survived had diagnosis code 862.8 (Injury to multiple and unspecified intrathoracic organs, without mention of open wound into cavity). About 20% of patients who survived had diagnosis code 861.13 (Laceration of heart with penetration of heart chambers with open wound into thorax). About 20% of patients who survived had diagnosis code 929.9 (Crushing injury of unspecified site). As a result, some patients were erroneously given an AIS of 6. The issue of overestimation in STATA-ICDPIC program has been reported in previous studies [15]. In addition, the poor performance of ICDPIC could be attributed to misclassifications in ICD-9 coding. Spinal column lesions/fractures were poorly captured in ICD-9-CM discharge codes [16,17], frequently appearing in the medical discharge abstract even when the injury was chronic, rather than acute. Misclassifications in ICD-9 coding could be caused by many factors, including coders’ inadequate training and experience, lack of facility quality-control efforts, and coders’ unintentional and intentional errors [18]. Higher quality ISS scores can be obtained by having two coders independently assign ISS scores. A third coder may be needed to address any substantial disagreement between those two coders. The inter-rater reliability can be assessed by calculating intra-class correlation. However, this approach of ISS scoring is not always feasible in trauma studies, especially when using existing national survey data. Computer-based ISS scoring has been proved to be useful when AIS scores are not available in the source data [19].

Though the survival rate of patients with an ISS = 75 could be overestimated in our study, these patients should not be assumed to have unsurvivable injuries. Trauma studies that deliberately excluded patients with an ISS = 75 may be subject to biases [5–9]. Studies that evaluated the effects of interventions on improving trauma outcomes were at the highest risk of yielding biased results. Since patients with an ISS = 75 sustained the most severe injuries, interventions that performed well among patients with an ISS <75 might not be effective among patients with an ISS = 75. Therefore, by excluding patients with an ISS = 75, the effectiveness of those interventions may have been overestimated. Studies that assessed the performance of injury severity scales might have obtained inaccurate estimates. They excluded patients with an ISS = 75 because they believed that cohorts with a large number of very severe cases could dilute the discriminative power of injury severity scales. However, it would be arbitrary to assume that all patients with an ISS = 75 had very severe injuries without examining the approach used to generate the ISS scores and the actual mortality status of these patients. These studies would have lost power of analysis if a significant proportion of the patients with an ISS = 75 actually had non-lethal injuries. Studies that examined the risk factors associated with increased trauma mortality were at the lowest risk of obtaining biased results. This was because risk factors that were found to be associated with increased trauma mortality among patients with an ISS<75 were likely to contribute to increased trauma mortality among patients with an ISS = 75. However, the inclusion of patients with an ISS = 75 would not affect the study findings. The rationale for deliberately excluding these patients is not always justified.

Trauma researchers should be very cautious when considering excluding patients with an ISS = 75 from analysis. To avoid potential biases, trauma researchers should always examine the mortality status of patients with an ISS = 75 when deciding whether these patients should be excluded. Because the approach used to generate ISS scores may affect the findings in trauma studies, researchers should explicitly describe the approach used to generate ISS scores and discuss the limitations of using that approach. If the AIS/ISS scores are mapped from ICD-9-CM diagnosis codes using a computer program, the limitations of using computer-assigned ISS score should be discussed. If the AIS/ISS scores are assigned by trained coders, errors in using human-assigned ISS scores should be explained, such as misspecification, miscoding, and upcoding [20].

It appeared that the mortality risks of patients with an ISS = 75 did not vary significantly across different levels of trauma centers (15.6% vs. 13.0%, P = 0.16) (Table 2). These results should be interpreted with caution. The adjustment for patient profiles using advanced statistical methodology is needed when making mortality risk comparisons across different levels of trauma centers. We did not conduct mortality risk comparisons because the focus of this study was to examine the characteristics and hospital disposition of patients with an ISS = 75. Further research is warranted to examine whether trauma centers achieve better outcomes among patients with an ISS = 75 compared to non-trauma hospitals.

## Conclusions

Almost half of patients with an ISS = 75 survived their injuries. Patients with an ISS = 75 should not be assumed to have unsurvivable injuries, and trauma researchers should be very cautious when considering excluding patients with an ISS = 75 from analysis. To avoid potential biases, researchers should always examine the mortality status of patients with an ISS = 75 when considering excluding these patients from analysis. Whether trauma centers achieve better outcomes than non-trauma hospitals among patients with an ISS = 75 warrants further research.

## Supporting Information

S1 Appendix Life-threatening injuries defined by the American College of Surgeon Committee on Trauma (ACS-COT). (DOCX)
