# Supplementary material for: Trauma with Injury Severity Score of 75: Are These Unsurvivable Injuries?
Source: PLoS One. 2015 Jul 31;10(7):e0134821. doi: 10.1371/journal.pone.0134821 (PMC4521713; doi:10.1371/journal.pone.0134821)
Supplement: S1 Appendix — (DOCX) [file pone.0134821.s001.docx]

**S1 Appendix:** Life-threatening injuries defined by the American College of Surgeon Committee on Trauma (ACS-COT)^1^

| ACS-COT Description of Life-Threatening or Critical Injury | Assumption | *ICD-9-CM* Diagnostic Codes |
| --- | --- | --- |
| Injury to aorta, carotid, and vertebral vessels |  | 900, 901 |
| Injury to heart |  | 861.0, 861.1 |
| >2 Rib fractures or bilateral pulmonary  contusions | >2 Rib fractures, flail chest, or pulmonary contusions | 807.03-807.09, 807.4, 861.20-861.22,  861.30-861.32 |
| Injury to abdominal vasculature |  | 902 |
| Open fracture with loss of distal pulses | Complete or partial amputation of upper or lower extremity | 887, 897 |
| Open skull fracture |  | 800.5-800.9, 801.5-801.9, 804.5-804.9 |
| GCS score <14 | Cerebral laceration with moderate to severe loss of consciousness | 851.03-851.05, 851.13-851.15, 851.23-851.25, 851.33-851.35, 851.43-851.45,  851.53-851.55, 851.63-851.65,  851.73-851.75, 851.83-851.85,  851.93-851.95 |
|  | Subarachnoid, subdural, or epidural hemorrhage with moderate to severe loss of  consciousness | 852.03-852.05, 852.13-852.15, 852.23-852.25, 852.33-852.35, 852.43-852.45,  852.53-852.55 |
|  | Other intracranial hemorrhage with moderate to severe loss of consciousness | 853.03-853.05, 853.13-853.15 |
|  | Intracranial injury of other and unspecified nature with moderate to severe loss of consciousness | 854.03-854.05, 854.13-854.15 |
| Any vertebral column fracture | Fracture of vertebral column | 805, 806 |
| Open fracture of long bone | Open fracture of humerus, radius or ulna, femur, tibia, fibula | 812.1, 812.3, 812.5, 813.1, 813.3, 813.5, 813.8, 820.1, 820.3, 820.9, 821.1, 821.3, 823.1, 823.3, 823.9 |
| Severe torso injury with comorbid disease | Injury to thoracic organs, GI tract, liver, spleen, kidney, pelvic organs, other intra-abdominal  organs, unspecified intra-abdominal organs | 862-869 with comorbid disease as defined by Elixhauser et al^2^ |
| Grade IV liver laceration with >6 U of PRBCs transfused | Did not include |  |
| Pelvic fracture with >6 U of PRBCs transfused | Did not include |  |

Abbreviations: ACS-COT, American College of Surgeons Committee on Trauma; GCS, Glasgow Coma Scale; GI, gastrointestinal; *ICD-9-CM*, *International Classification of Diseases, Ninth Revision, Clinical Modification*; PRBCs, packed red blood cells.

1. This table is cited from: Mohan D, Rosengart MR, Farris C, Cohen E, Angus DC, Barnato AE. Assessing the Feasibility of the American College of Surgeons' Benchmarks for the Triage of Trauma Patients. Arch Surg. 2011;146(7):786-92.

2. Elixhauser A, Steiner C, Harris DR, Coffey RM. Comorbidity measures for use with administrative data. Med Care. 1998;36(1):8-27.
